# Demonstration of the Nonlocal Josephson Effect in Andreev Molecules

**DOI:** 10.1021/acs.nanolett.3c02066

**Published:** 2023-08-08

**Authors:** Daniel Z. Haxell, Marco Coraiola, Manuel Hinderling, Sofieke C. ten Kate, Deividas Sabonis, Aleksandr E. Svetogorov, Wolfgang Belzig, Erik Cheah, Filip Krizek, Rüdiger Schott, Werner Wegscheider, Fabrizio Nichele

**Affiliations:** † IBM Research Europe−Zurich, Säumerstrasse 4, 8803 Rüschlikon, Switzerland; ‡ Fachbereich Physik, Universität Konstanz, D-78457 Konstanz, Germany; ¶ Solid State Physics Laboratory, ETH Zürich, 8093 Zürich, Switzerland

**Keywords:** Hybrid materials, superconductor-semiconductor, Andreev bound state, Andreev molecule, φ_0_-junction

## Abstract

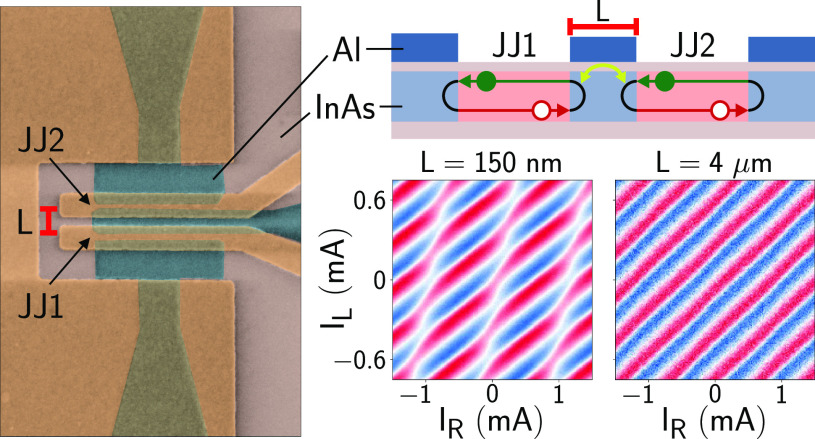

We perform switching current measurements of planar Josephson junctions (JJs) coupled by a common superconducting electrode with independent control over the two superconducting phase differences. We observe an anomalous phase shift in the current–phase relation of a JJ as a function of gate voltage or phase difference in the second JJ. This demonstrates the nonlocal Josephson effect, and the implementation of a φ_0_-junction which is tunable both electrostatically and magnetically. The anomalous phase shift is larger for shorter distances between the JJs and vanishes for distances much longer than the superconducting coherence length. Results are consistent with the hybridization of Andreev bound states, leading to the formation of an Andreev molecule. Our devices constitute a realization of a tunable superconducting phase source and could enable new coupling schemes for hybrid quantum devices.

The development of high-quality hybrid superconductor–semiconductor materials over the past decade enabled new possibilities in superconducting electronics and quantum computing.^1−3^ In particular, Andreev bound states (ABSs)^4−6^ arising in superconductor–semiconductor–superconductor Josephson junctions (JJs)^7−10^ offer functionalities not attainable in metallic JJs. A prominent example is the electrostatic tuning of the critical current,^3,11,12^ which allows for JJ field-effect transistors,^13−16^ voltage-tunable superconducting qubits,^17−20^ resonators,^21,22^ and amplifiers.^23−25^ Moreover, the interplay between ABSs, spin–orbit interaction, and Zeeman fields results in nonreciprocal switching currents^26−29^ and anomalous phase offsets, or φ_0_-junctions,^30−38^ with applications in superconducting electronics and spintronics.^39^ A yet largely unexplored possibility offered by superconductor–semiconductor hybrids is the engineering of Andreev molecules from the hybridization of spatially overlapping ABSs.^40−43^ Predicted to arise in JJs coupling over length scales comparable to the superconducting coherence length, Andreev molecules offer a promising platform to realize φ_0_-junctions^40^ and novel manipulation and coupling schemes for Andreev qubits.^41^ Experimental studies of ABS hybridization focused on two-terminal quantum dots^44,45^ and quantum dot chains.^46^ Recently, engineering of Andreev molecules was demonstrated in open, multiply connected geometries^47^ and laterally coupled JJs.^48,49^ Measurements of the switching current in double InAs nanowires revealed a nonlocal Josephson effect,^48^ however the device geometry did not allow measurements of phase shifts in the current–phase relation (CPR).

Here we demonstrate the generation and electrical tuning of an anomalous phase shift in planar JJs that share a mesoscopic superconducting electrode. Inspired by ref (40), we realized devices consisting of two JJs sharing a common electrode and embedded in a superconducting double-loop geometry. The expected phase anomaly was ascribed^42^ to the interplay between two distinct Cooper pair transfer mechanisms at φ_1_ = φ_2_ and at φ_1_ = −φ_2_, namely double crossed Andreev reflection (dCAR) and double elastic cotunneling (dEC), respectively. Different from the proposal of ref (42), which considered semiconductor nanowires, we used planar JJs containing several ABSs. Such devices are lithographically defined with a top-down approach and, by tuning their geometry, allow for large switching currents.

The double-loop geometry allowed us to independently tune the superconducting phase differences φ_1_ and φ_2_ across the two JJs, named JJ1 and JJ2, respectively. Furthermore, it allowed us to characterize the CPR of JJ1 for different values of φ_2_. A coupling between JJ1 and JJ2 manifested as a distorted and phase-shifted CPR of JJ1 for φ_2_ ≠ (0, π). Such a coupled system realizes the nonlocal Josephson effect: an anomalous phase shift (or, equivalently, an anomalous supercurrent at zero phase difference) was nonlocally induced in JJ1 by the phase difference across JJ2. Varying the length *L* of the common superconducting electrode, we observed that the phase shift was larger for small *L*, and it vanished for *L* much longer than the superconducting coherence length. Our observations are consistent with the hybridization of ABSs originating from the two JJs, resulting in the formation of an Andreev molecule. As the superconducting phase offset is electrically tuned by a current flowing in a flux line or a voltage applied to a gate, our devices constitute a demonstration of a tunable superconducting phase source. Our findings open up new avenues for the design and implementation of advanced nanoscale quantum devices with enhanced controllability and functionality.

Experiments were performed on four devices (Devices 1 to 4) defined in the same epitaxial heterostructure of InAs and Al,^3,50^ measured in a dilution refrigerator with a base temperature below 10 mK. Figure 1a shows a false-colored scanning electron micrograph of Device 1, indicating the exposed InAs (pink), the epitaxial Al (blue), the gate electrodes (yellow), and the flux-bias lines (purple). Devices consisted of a small superconducting loop, threaded by flux Φ_R_, embedded in the arm of a large superconducting loop, threaded by flux Φ_L_. The region where the two loops merged (bottom right in Figure 1a) is shown in Figure 1b. Here, three Al leads defined two nominally identical JJs that shared a central Al electrode of length *L*. We label the bottom and top junctions in Figure 1b JJ1 and JJ2, respectively. In each JJ, the width of the Al electrodes was 800 nm and the length of the junction was 40 nm. From the junction geometry, we estimate between 40 to 100 transverse modes to be present, for typical values of electron sheet density (see Supporting Information for details). Both JJ1 and JJ2 were controlled by gate electrodes, energized by voltages of *V*_1_ and *V*_2_, respectively. A third gate, at voltage *V*_3_, was set to −3 V throughout the experiment to prevent parallel conducting paths. A narrow Al constriction was defined on the left arm of the large Al loop (Figure 1a). This constriction limited the maximum supercurrent flowing in the device, making switching current measurements feasible without warming up the apparatus. Devices 1 to 3 differed exclusively by the parameter *L*, which was 150, 400, and 4000 nm, respectively. Device 4 was lithographically identical with Device 1 and is presented in the Supporting Information, together with further details on the heterostructure and sample fabrication.

**Figure 1:**
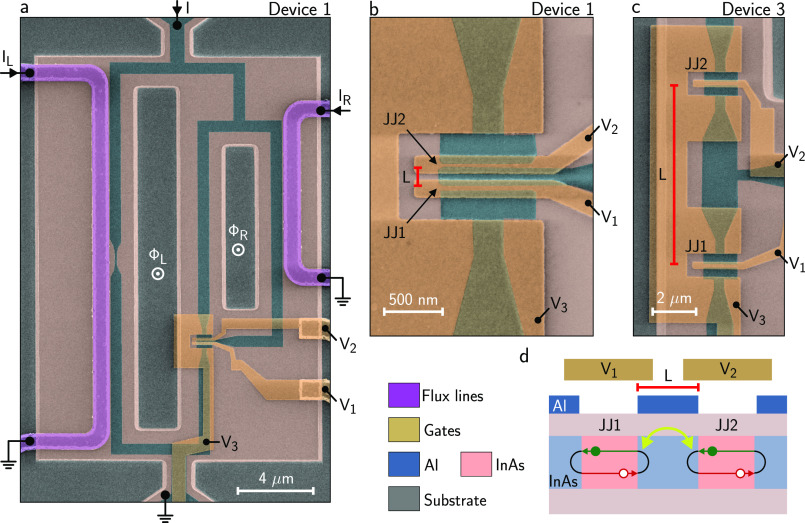
Devices under study and measurement setup. (a) False-colored scanning electron micrograph of a sample identical with Device 1, together with measurement schematics. The substrate is shown in gray, the exposed III–V semiconductor in pink, the epitaxial Al in blue, gates in yellow and flux lines in purple. (b) Zoom-in of panel a around the Josephson junctions (JJs). The distance between the junctions is *L* = 150 nm. (c) Similar to panel b but for Device 3, which has *L* = 4 μm. (d) Schematic cross-section (not to scale) of the JJs sharing a common electrode of length *L*. Andreev bound states originating from the two JJs, spatially extended over distances in excess of *L*, overlap and hybridize, forming an Andreev molecule.

The measurement setup used to measure the switching currents is schematically depicted in Figure 1a. After compensation for a global magnetic field offset using a vector magnet, local magnetic fluxes were generated by applying slowly varying currents *I*_L_ and *I*_R_ in the flux lines. Switching currents of the entire device were obtained by ramping the current *I* from zero to around 35 μA (depending on the device) with a repetition rate of 133 Hz and detecting when the four-terminal voltage drop *V* exceeded a threshold. As planar devices are characterized by an intrinsically large spread of their switching current,^51^ we averaged the results over 16 ramps for each data point. In all of our devices, JJ1 had a maximum supercurrent of approximately 450 nA, while the Al constrictions consistently showed switching currents of *I*_Al_ ∼ 34 μA, independent of Φ_L_ and Φ_R_. Due to the large asymmetry between the arms of the device, the switching current *I*_SW_ of the right arm was obtained by subtracting *I*_Al_ from the switching current of the entire device. Further details on the measurement setup are discussed in the Supporting Information.

Our devices allowed an independent tuning of the phase differences across the two junctions. The phase difference φ_1_ across JJ1 was tuned by the magnetic flux impinging within the perimeter of the device as φ_1_ = 2π(Φ_L_ + Φ_R_)/Φ_0_, where Φ_0_ is the superconducting magnetic flux quantum. The phase difference φ_2_ across JJ2 was instead tuned by the magnetic flux Φ_R_. However, as JJ2 was bypassed by a large stripe of Al, φ_2_ was not expected to affect *I*_SW_, unless a nonlocal Josephson effect was present; in the absence of coupling between JJ1 and JJ2, *I*_SW_ would simply represent the CPR of JJ1.

We further note that the finite geometric and kinetic inductances of the superconducting loops were too small to significantly distort the CPR of JJ1 and JJ2. Similarly, coupling between the two loops mediated by shared inductance was negligible. The inductances of the inner and outer loops were 105 pH and 200 pH, respectively, while their common Al segment (right side of Figure 1a) had an inductance of 58 pH. Such values are significantly smaller than the Josephson inductances of JJ1 and JJ2, which were always greater than 800 pH. The absence of inductive coupling was experimentally confirmed by results obtained on Device 3, as discussed below.

Figure 2a shows the switching current *I*_SW_ of Device 1 as a function of *I*_L_ and *I*_R_ performed with *V*_1_ = 0 and *V*_2_ = 0 (both JJs open). Clear supercurrent oscillations were present, which depended on both *I*_L_ and *I*_R_. The vector Φ_L_ + Φ_R_ in Figure 2a shows the direction over which φ_1_ is expected to be maximally modulated. The vectors Φ_R_ – Φ_L_ and Φ_L_ indicate the directions over which φ_1_ and φ_2_ are constant, respectively. Details of the definition of such vectors are presented in the Supporting Information. Clear modulations of the supercurrent were observed along Φ_R_ – Φ_L_, confirming a coupling between JJ1 and JJ2. Figures 2b and 2c show equivalent measurements performed after setting *V*_2_ = −1.2 V and *V*_2_ = −2.4 V, respectively. As *V*_2_ was set more negative, JJ2 was depleted, and the supercurrent oscillations became more and more regular, until supercurrent modulations were completely suppressed along Φ_R_ – Φ_L_ (Figure 2c) and JJ1 displayed a conventional forward-skewed CPR. Further, we note that *V*_2_ did not alter the maximum switching current amplitude, confirming the absence of trivial electrostatic coupling between the gate of JJ2 and JJ1. Supercurrent measurements for Device 2, which had *L* = 400 nm, are shown in Figures 2e–g for *V*_1_ = 0 and varying *V*_2_. Anomalous phase modulations were still present in the supercurrent oscillations of Figure 2e, despite being significantly weaker than those in Figure 2a. Setting *V*_2_ = −2 V (Figure 2g) suppressed any remaining phase modulation, resulting in a conventional CPR as in Figure 2c. Finally, Figures 2d and 2h show measurements performed in Device 3, with *L* = 4 μm. In this case, phase modulations never occurred along Φ_R_ – Φ_L_, neither for *V*_2_ = 0 (Figure 2d) nor when *V*_2_ = −3 V (Figure 2h), demonstrating the absence of coupling in well-separated JJs and that contributions of loop inductance and circulating currents to phase shifts were negligible.

**Figure 2:**
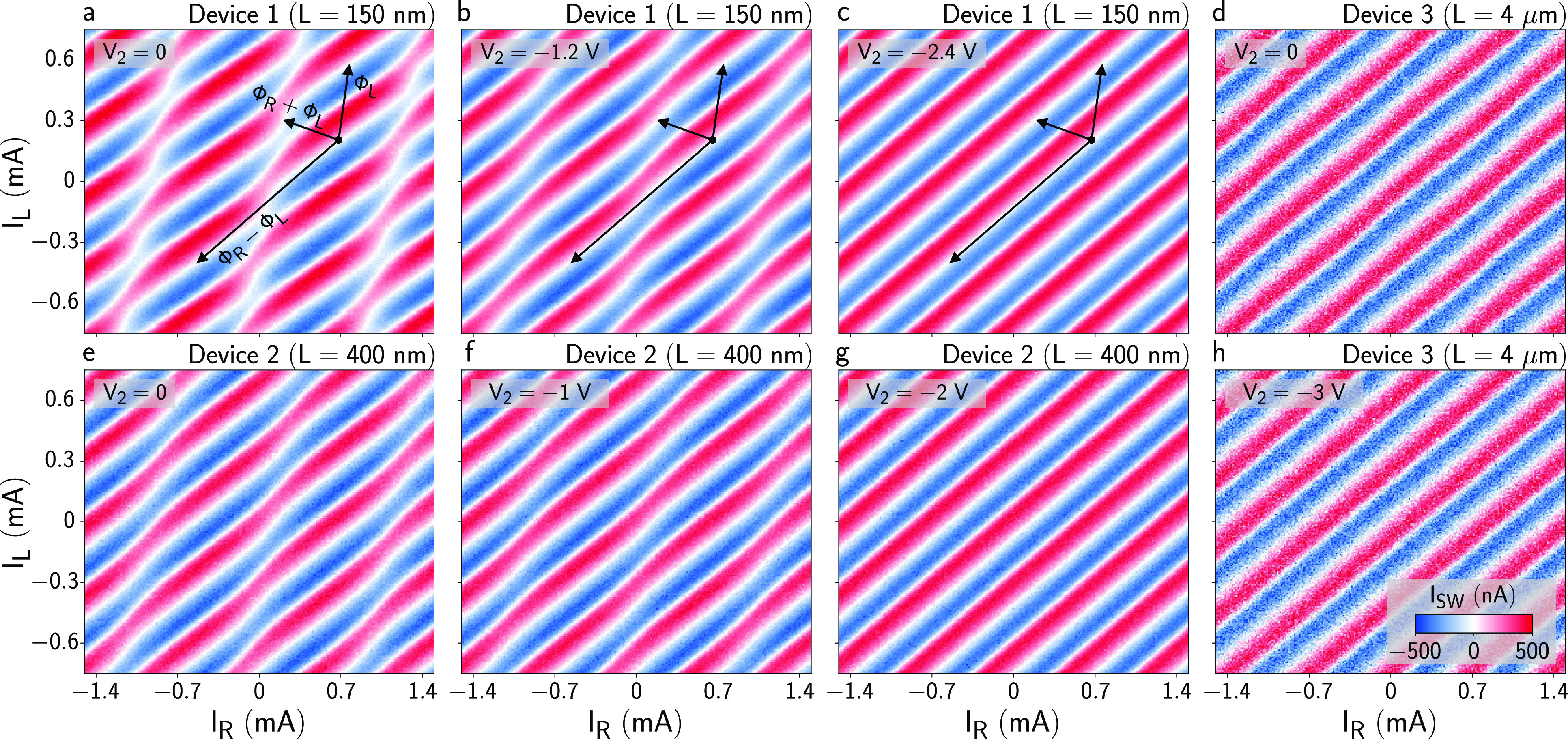
Phase-dependent supercurrent and evidence of nonlocal Josephson effect. (a–c) Switching current *I*_SW_ for Device 1 after subtraction of the switching current of the Al constriction *I*_Al_, for *V*_1_ = 0 and *V*_2_ = 0, – 1.2, and −2.4 V, respectively. Black arrows indicate the direction Φ_L_ + Φ_R_, which is the direction of maximal modulation of φ_1_, Φ_R_ – Φ_L_, which is the direction over which φ_1_ is constant, and Φ_L_, which is the direction over which φ_2_ is constant. (d) *I*_SW_ in Device 3 for *V*_1_ = *V*_2_ = 0. (e–g) Same as panels a–c, but for Device 2. (h) *I*_SW_ in Device 3 for *V*_2_ = −3 V.

Figure 3 presents the dependence of supercurrent oscillations in Device 1 on *V*_1_ and *V*_2_. Panel a shows supercurrent oscillations for *V*_1_ = −1.4 V and *V*_2_ = 0: while the oscillation amplitude was reduced by setting *V*_1_ negative, the oscillation pattern was almost identical with that of Figure 2a, indicating that *V*_1_ had a negligible influence on the anomalous phase shift. The effect of gate voltages was further investigated by measuring supercurrents along the paths γ_1_ and γ_2_, shown as arrows in Figure 3a. Figure 3b shows the CPR of JJ1 measured along γ_1_ with *V*_2_ = −2 V, that is, with no current flowing in JJ2. Selected linecuts are plotted in Figure 3c. Again, we note that *V*_1_ did not induce a phase shift but simply decreased the oscillation amplitude until no current flowed in the right arm of the device. The linecuts in Figure 3c demonstrate that JJ1 had a forward-skewed CPR, which can be parametrized by an effective junction transmission of τ̅ = 0.80, indicating the presence of highly transmissive ABSs in JJ1 (see Supporting Information for details). Figures 3d and 3e show the CPR of JJ1 along γ_2_ and γ_1_, respectively, measured for *V*_1_ = 0. Selected linecuts of Figure 3e are shown in Figure 3f. Both Figures 3d and 3e show distorted and phase-shifted supercurrent oscillations that evolved with decreasing *V*_2_, saturating to a conventional forward-skewed CPR for *V*_2_ ≲ −2 V.

**Figure 3:**
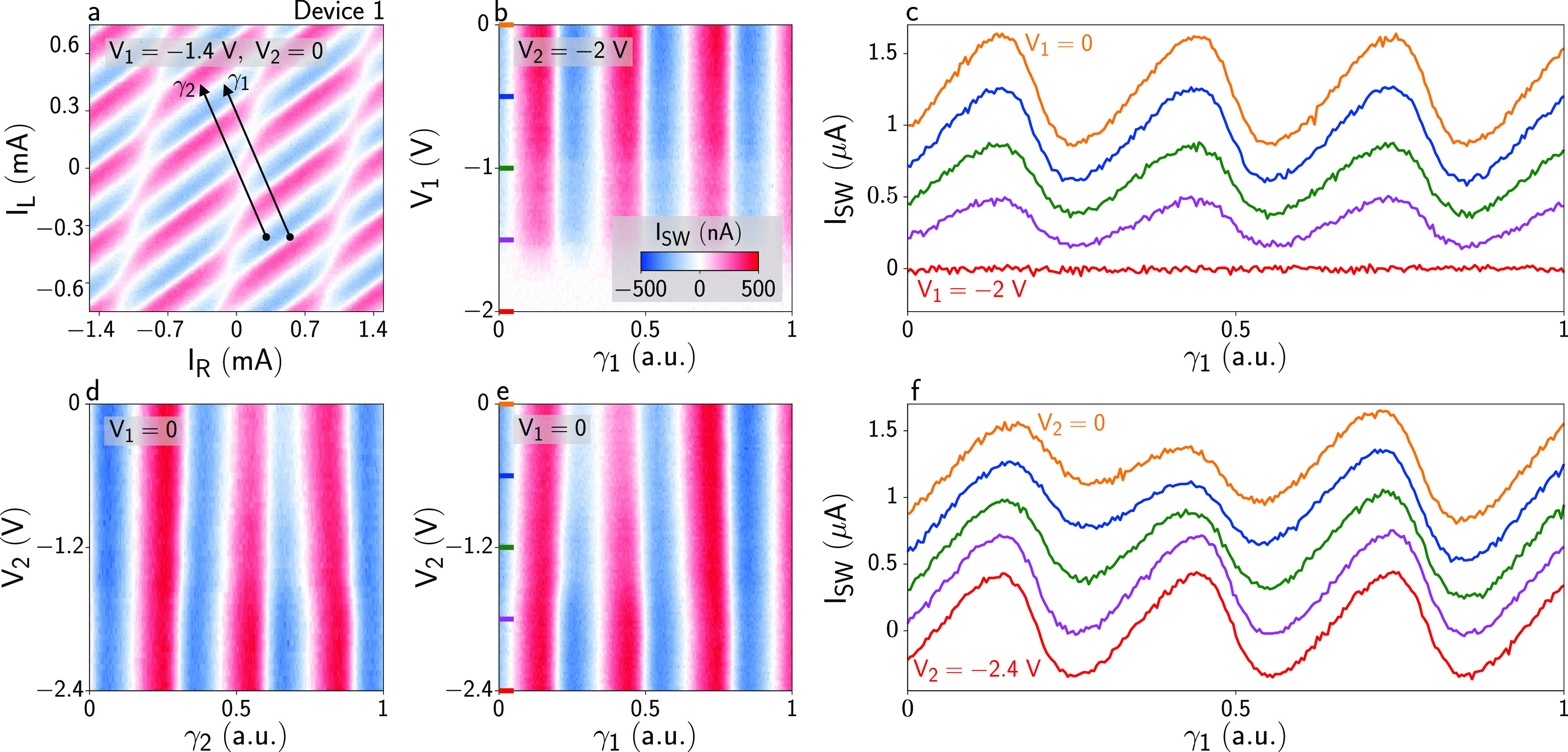
Gate-dependence of switching currents and nonlocal Josephson effect. (a) Switching current *I*_SW_ for Device 1 as a function of Φ_L_ and Φ_R_, measured with *V*_1_ = −1.4 V and *V*_2_ = 0. The paths γ_1_ and γ_2_ are shown as black arrows. (b) *I*_SW_ as a function of *V*_1_ and γ_1_, measured with *V*_2_ = −2 V. In this configuration, no current flows in JJ2 and *I*_SW_ reflects the current–phase relation of JJ1 without hybridization. (c) Linecuts of *I*_SW_ extracted from panel b for various values of *V*_1_ (see markers in panel b). (d) *I*_SW_ as a function of *V*_2_ and γ_2_, measured with *V*_1_ = 0. In this configuration, JJ1 was completely open. (e) As in panel d but measured along γ_1_. (f) Linecuts of *I*_SW_ extracted from panel e for various values of *V*_2_ (see markers in part e).

Figure 4 summarizes the main results of this work. After performing an appropriate basis transformation to the data in Figures 2a–c, it is possible to display *I*_SW_ as a function of φ_1_ for selected values of φ_2_ (see Supporting Information for details). For example, Figure 4a shows *I*_SW_(φ_1_) for φ_2_ = 0.8π, that is, when the phase shift was found to be the largest. Figure 4a also highlights the quantities Δ*I*_1_ and Δφ_1_, which represent the anomalous supercurrent (i.e., the supercurrent at φ_1_ = 0) and anomalous phase shift, respectively. The dependence of Δ*I*_1_ and Δφ_1_ on φ_2_ is further shown in Figures 4b and 4c. Similar results for Device 2 are shown in Figure 4d–f. The data presented here demonstrates coupling between the JJs, consistent with the formation of an Andreev molecule from the ABSs of JJ1 and JJ2.^40^ Time-reversal symmetry requires *I*_1_(−φ_1_, – φ_2_) = −*I*_1_(φ_1_, φ_2_), which imposes Δφ_1_ = 0 at φ_2_ = (0, π). This condition was used to accurately account for a constant offset to Δφ_1_. For φ_2_ ≠ (0, π), the CPR of JJ1 gained an anomalous phase shift Δφ_1_ or, equivalently, an anomalous supercurrent Δ*I*_1_ at φ_1_ = 0, controlled by both phase and gate tuning of JJ2. Both the anomalous phase shift and anomalous supercurrent were symmetric and periodic in φ_2_, consistent with theoretical expectations.^40^ The phase shift Δφ_1_ was also an odd function of φ_2_, compatible with the relative interplay of the dCAR and dEC processes as a function of the nonlocal phase. There was also a pronounced forward skewness toward Δφ_1_, which is a consequence of the nonsinusoidal CPR of both JJs. The largest Δφ_1_ measured in Device 1 was ±0.22π, which resulted in Δ*I*_1_ = ± 170 nA. These values might be increased further in devices with shorter *L*. We confirmed that coupling takes place over length scales of at least 400 nm but significantly smaller than 4 μm. Such length scales are consistent with those of superconducting correlations in our devices. In the InAs quantum well, we expect a superconducting coherence length ξ_InAs_ ∼ 600 nm, compared to ξ_Al_ ∼ 100 nm of the Al film^52^ (see Supporting Information for calculation of ξ_InAs_). These length scales are compatible with the absence of coupling in Device 3 (*L* = 4 μm), and they indicate that both the semiconductor and the superconductor might be relevant to mediating the coupling over short JJ separations. Extending our experiments to several devices with varying *L* values will make it possible to extract the typical length scale governing the nonlocal effect, thus providing a better understanding of the microscopic coupling mechanisms.

**Figure 4:**
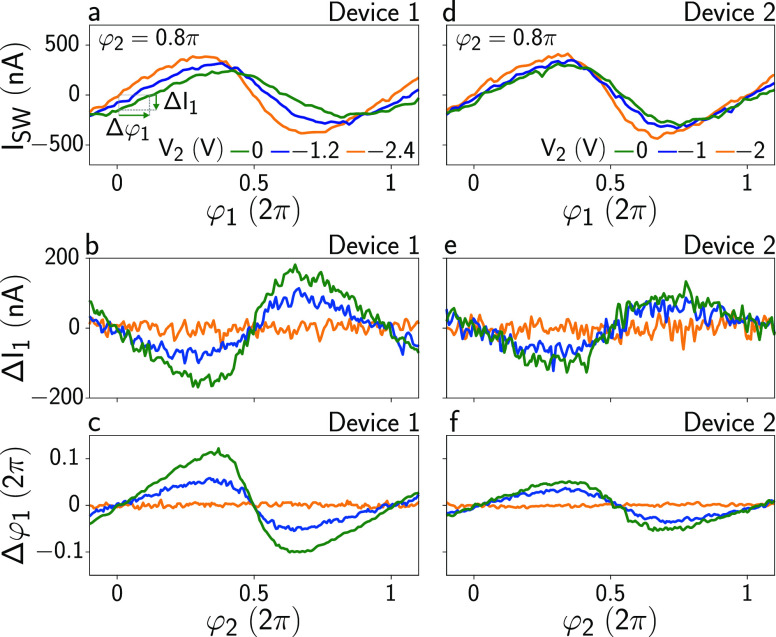
Anomalous supercurrent and anomalous phase shift. (a) Switching current *I*_SW_ in Device 1 as a function of φ_1_ measured for φ_2_ = 0.8π at three values of *V*_2_. Quantities Δ*I*_1_ and Δφ_1_ are defined. More details on the data analysis required to produce this plot are presented in the Supporting Information. (b) Anomalous supercurrent Δ*I*_1_ as a function of φ_2_ for three values of *V*_2_ (see legend in part a). (c) Anomalous phase shift Δφ_1_ as a function of φ_2_ for three values of *V*_2_ (see legend in panel a). (d–f) As parts a–c, but for Device 2.

Further insights into the nonlocal Josephson effect are gained via the gate dependence shown in Figure 3. Data indicate that *V*_1_ affected the amplitude of *I*_SW_ but not Δφ_1_, while *V*_2_ tuned Δφ_1_ without any influence on the amplitude of *I*_SW_. A gate-dependent phase shift can result from tuning of the transmission and number of ABSs in each JJ, affecting the degree of hybridization between ABSs in JJ1 and JJ2. If the gate tuning of Δφ_1_ was only due to a change of the ABS transmissions as a function of *V*_2_, we would expect the effect to be symmetric with respect to JJ1 and JJ2, contrary to the observations. Previous work also showed that gate voltages mainly affect hybrid JJs by reducing the number of ABSs, not by changing the ABS transmission.^53^ We instead speculate that the different dependences of *I*_SW_ on *V*_1_ and *V*_2_ reflect how Δφ_1_ is affected by the number of ABSs present in each junction. In the present case, *V*_2_ controls Δφ_1_ by tuning the number of ABSs in JJ2 available for hybridization with the ABSs in JJ1. As JJ2 is depleted, ABSs in JJ1 progressively lose states of JJ2 to hybridize with, until a conventional CPR is restored. On the other hand, setting *V*_1_ negative decreases the number of ABSs in JJ1, which directly results in a decrease of *I*_SW_. However, as long as the number of ABSs in JJ2 is unchanged, the hybridization of the remaining ABSs in JJ1 is also unchanged, and Δφ_1_ remains constant.

Previous work demonstrated φ_0_-junction behavior in the same material system by the combination of spin–orbit coupling and external magnetic fields.^37^ To achieve similar Δφ_1_ as in Figure 4c, in-plane magnetic fields of approximately 150 mT were required. Such magnetic fields are hardly compatible with existing superconducting electronics, making the nonlocal Josephson effect mediated by Andreev molecules a particularly promising avenue to generate arbitrary phase shifts in superconducting devices. Another recent work showed a persistent φ_0_-junction behavior induced by ferromagnetic elements in a JJ and field cycling, constituting a phase battery;^38^ the φ_0_-junction that we realized here is instead continuously and electrically tunable over short time scales; hence, it could be promptly utilized as a phase source for applications in superconducting electronics and spintronics.

In conclusion, our investigation of phase-tunable JJs placed in close proximity to each other and sharing a common electrode demonstrated the formation of an Andreev molecule exhibiting a nonlocal Josephson effect. This manifested as an anomalous phase shift and an anomalous supercurrent arising in one JJ, depending on phase tuning and gating of the second JJ. In light of our results, Andreev molecules expand the available toolset of functionality in hybrid materials, also enabling new quantum manipulation schemes and coupling architectures for hybrid qubits.

## Supporting Information Available

Detailed discussion on sample fabrication and electrical measurements; calculations of the superconducting coherence length, loop inductances, and the effective junction transmission; details on current-to-flux conversion; and additional experimental results on Device 4, which was identical with Device 1 (PDF)

## Notes

During the final preparation of this manuscript, a paper reporting similar effects was posted online.^54^
