# Supplementary material for: Demonstration of the Nonlocal Josephson Effect in Andreev Molecules
Source: Nano Lett. 2023 Aug 8;23(16):7532–8. doi: 10.1021/acs.nanolett.3c02066 (PMC10450812; doi:10.1021/acs.nanolett.3c02066)
Supplement: Supplementary file 1 — nl3c02066_si_001.pdf [file nl3c02066_si_001.pdf]

Supporting Information: Demonstration of the Nonlocal Josephson Effect in Andreev Molecules

Daniel Z. Haxell,[†,§] Marco Coraiola,[†,§] Manuel Hinderling,[†] Sofieke C. ten Kate,[†] Deividas Sabonis,[†] Aleksandr E. Svetogorov,[‡] Wolfgang Belzig,[‡] Erik Cheah,[¶] Filip Krizek,[†,¶,‖] Rüdiger Schott,[¶] Werner Wegscheider,[¶] and Fabrizio Nichele[*,†]

†*IBM Research Europe—Zurich, Säumerstrasse 4, 8803 Rüschlikon, Switzerland*

‡*Fachbereich Physik, Universität Konstanz, D-78457 Konstanz, Germany*

¶*Solid State Physics Laboratory, ETH Zürich, 8093 Zürich, Switzerland*

§*These authors contributed equally.*

‖*Present address: Institute of Physics, Czech Academy of Sciences, 162 00 Prague, Czech Republic*

E-mail: fni@zurich.ibm.com

Materials and Methods

The heterostructure used in this work was grown with molecular beam epitaxy techniques on an InP (001) substrate. The top part of the heterostructure consisted of a step-graded InAlAs buffer, and an 8 nm thick InAs quantum well confined between two $In_{0.75}Ga_{0.25}As$ barriers. The bottom and top barriers were 6 nm and 13 nm thick, respectively. On top of the III–V stack, two monolayers of GaAs and a 15 nm thick Al layer were deposited *in situ*, without breaking vacuum. Characterization of the 2DEG in a Hall bar geometry revealed a peak mobility of 18×10^3 cm^{2}V^{-1}s^{-1} at an electron sheet density of 8×10^{11} cm^{-2}. This resulted in an electron mean free path $l_e \gtrsim 260$ nm, indicating that Josephson junctions in

our devices were in the ballistic regime. The superconducting coherence length in InAs was calculated as $\xi_{\mathrm{InAs}} = \sqrt{\hbar v_{\mathrm{F}} l_{\mathrm{e}}/(2\Delta^*)} = 600$ nm. Here $\hbar$ is the reduced Plank constant, v_{F} is the electron Fermi velocity, and $\Delta^* = 180\ \mu$eV is the induced superconducting gap in InAs, which we consider similar to that of bulk Al. Fabrication of the devices was conducted in an identical manner to that described in Ref. 1.

The Al film had a kinetic inductance of 1.7 pH per unit square, calculated from measurements in a Hall bar geometry in the same material.[1,2] The geometric and kinetic inductance contributions are calculated for the outer [$\Phi_{\mathrm{L}} + \Phi_{\mathrm{R}}$ in Fig. 1(a) of the Main Text] and inner [Φ_{R} in Fig. 1(a) of the Main Text] loops. The values for the outer (inner) loop were 30 pH (15 pH) for the geometric inductance and 170 pH (90 pH) for the kinetic inductance. The portion of the circuit shared by both loops, corresponding to the right branch in Fig. 1(a) of the Main Text, had a kinetic inductance of 50 pH and a geometric inductance of 8 pH.

Josephson junctions (JJs) were identical in design for all devices. From the junction geometry, the approximate number of transverse modes sustained by the junction is $N \approx W/(\lambda_{\mathrm{F}}/2)$, where λ_{F} is the Fermi wavelength. From measurements in a gated InAs Hall bar, the sheet carrier density is expected to vary between 4 and 22×10^{11} cm^{-2} for typical values of top-gate voltage. This gives a Fermi wavelength between 17 nm and 40 nm, implying a number of modes between 40 and 100. Measurements of the current–phase relation (CPR) in JJ1 of Device 1 [Fig. 3(b) of the Main Text] show a non-sinusoidal CPR, indicating the presence of highly transparent modes. The CPR at $V_1 = 0$ [see Fig. 3(c) of the Main Text] gives an effective junction transmission of $\bar{\tau} = 0.80$ over an effective number of highly-transmissive modes $\bar{N} = 16$.[3] For $V_1 > -1.5$ V, $\bar{\tau}$ was approximately constant.

Electrical measurements were performed in a dilution refrigerator with a mixing chamber base temperature below 10 mK. Electrical contacts to each device, except for the two flux lines, were provided by resistive looms with QDevil pi-filters at the mixing chamber level and RC filters at both mixing chamber and sample stage. The bias current I passing through the devices was sourced via a Keysight 33600 Waveform Generator. The two output

channels produced two synchronized and opposite voltage sawtooth waveforms with amplitude of about 6 V (depending on the specific device) and repetition rate of 133 Hz. The two waveforms were applied via two 163 kΩ resistors placed in series to device source and drain contact, resulting in a maximum current of approximately 35 μA. The voltage drop V across the device was measured in a four-terminal configuration via a home-made differential amplifier with a gain of 1000, a further amplification stage with gain of 42 provided by the internal amplifier of a Standford Research SR860 lock-in amplifier, and finally detected by a Keysight DSOX2024A oscilloscope. The oscilloscope measured the time needed for the voltage drop across the device to overcome a threshold, set at 7% of the maximum voltage measured in the resistive state. The switching time was averaged over 16 current ramps and converted into a current. With these measurement parameters, the transition from superconducting to resistive state was extremely sharp, making the exact choice of the experimental parameters irrelevant. Flux lines were connected via a superconducting loom, with pi-filters at the mixing chamber level to suppress high-frequency noise, resulting in a total line resistances below 5 Ω. Currents I_{L} and I_{R} were generated by two Yokogawa GS200 sources set to current mode. Low-pass RC filters with $R = 10$ kΩ and $C = 1$ μF were placed at the current source output.

Current-to-Phase Conversion

As described in the Main Text, currents I_{L} and I_{R} were injected into flux bias lines proximal to the device. Each current generated a magnetic field, predominantly impinging on the closest loop: I_{L} mainly controlled an external flux Φ_{L} threading the left loop, and I_{R} mainly controlled an external flux Φ_{R} through the right loop. Nevertheless, each flux line had a finite coupling to the furthest loop, meaning that Φ_{L} and Φ_{R} depended on both I_{L} and I_{R}. The phase difference across JJ1, φ_1, changed most strongly as a result of a flux threading the outer loop of the device [see Fig. 1(a) of the Main Text], which corresponds to $\Phi_{\mathrm{L}} + \Phi_{\mathrm{R}}$. This is

because a path along the outer loop contains only JJ1 (and the Al constriction), so the phase difference across JJ1 was proportional to the flux $\Phi_\mathrm{L} + \Phi_\mathrm{R}$ threading that area. We note that the arrow labeled $\Phi_\mathrm{L} + \Phi_\mathrm{R}$ in Fig. 2(a) of the Main Text corresponds to $\Phi_\mathrm{L} + \Phi_\mathrm{R} = \Phi_0$. The phase difference φ_1 was constant along the $\Phi_\mathrm{R} - \Phi_\mathrm{L}$ direction, since any increase in the flux through one loop was compensated by the flux through the other. We apply the same procedure to JJ2, showing that the phase φ_2 across JJ2 varied most strongly as a function of Φ_R and was constant along the Φ_L direction. We define the phase axes as those along which only one phase varies, meaning Φ_L corresponds to φ_1 and $\Phi_\mathrm{R} - \Phi_\mathrm{L}$ corresponds to φ_2. We therefore define these as our phase axes, and perform the conversion from $(I_\mathrm{L}, I_\mathrm{R})$ to (φ_1, φ_2) using the relation:

$$\begin{pmatrix} \varphi_1 \\ \varphi_2 \end{pmatrix} \equiv \frac{1}{\Phi_0} \begin{pmatrix} \Phi_\mathrm{L} \\ \Phi_\mathrm{R} - \Phi_\mathrm{L} \end{pmatrix} = \frac{1}{\Phi_0} \mathbf{M} \cdot \begin{pmatrix} I_\mathrm{L} \\ I_\mathrm{R} \end{pmatrix} = \frac{1}{\Phi_0} \begin{pmatrix} M_{11} & M_{12} \\ M_{21} & M_{22} \end{pmatrix} \cdot \begin{pmatrix} I_\mathrm{L} \\ I_\mathrm{R} \end{pmatrix}, \tag{1}$$

where $\Phi_0 = h/(2e)$ is the superconducting flux quantum and $\mathbf{M}$ is a matrix relating the flux line currents $(I_\mathrm{L}, I_\mathrm{R})$ to the fluxes $(\Phi_\mathrm{L}, \Phi_\mathrm{R} - \Phi_\mathrm{L})$. We calculate $\mathbf{M}$ for each device, using the switching current measurements taken at $V_1 = V_2 = 0$ [see Figs. 2(a) and (e) of the Main Text for Devices 1 and 2, respectively]. We evaluate Eq. 1 for $(\Phi_\mathrm{L}, \Phi_\mathrm{R} - \Phi_\mathrm{L}) = (\Phi_0, 0)$ and $(\Phi_\mathrm{L}, \Phi_\mathrm{R} - \Phi_\mathrm{L}) = (0, \Phi_0)$, and thereby obtain:

$$\mathbf{M} = \begin{pmatrix} 0.66 & -1.99 \\ 6.14 & -3.00 \end{pmatrix} \text{ pH} \tag{2}$$

for Device 1 and

$$\mathbf{M} = \begin{pmatrix} 0.75 & -2.05 \\ 6.28 & -3.10 \end{pmatrix} \text{ pH} \tag{3}$$

for Device 2. The good agreement between Eqs. 2 and 3 show that the loop sizes and the flux line fabrication was almost identical between Devices 1 and 2. The matrix $\mathbf{M}$ from Eq. 2 was used for Device 3, where independent evaluation was not possible due to the presence of

only one periodicity axes. The position of $\Phi_L = \Phi_R = 0$ was defined where the $I_{SW} = 0$ line intersected all flux periodicity axes, for increasing I_{SW} in the $\Phi_L + \Phi_R$ direction. Using the matrix $\mathbf{M}$ of Eq. 2 or 3, we apply the linear transformation of Eq. 1 to convert the (I_L, I_R) axes to (φ_1, φ_2). The result is plotted in Fig. S.1, for the data presented in Fig. 2 of the Main Text.

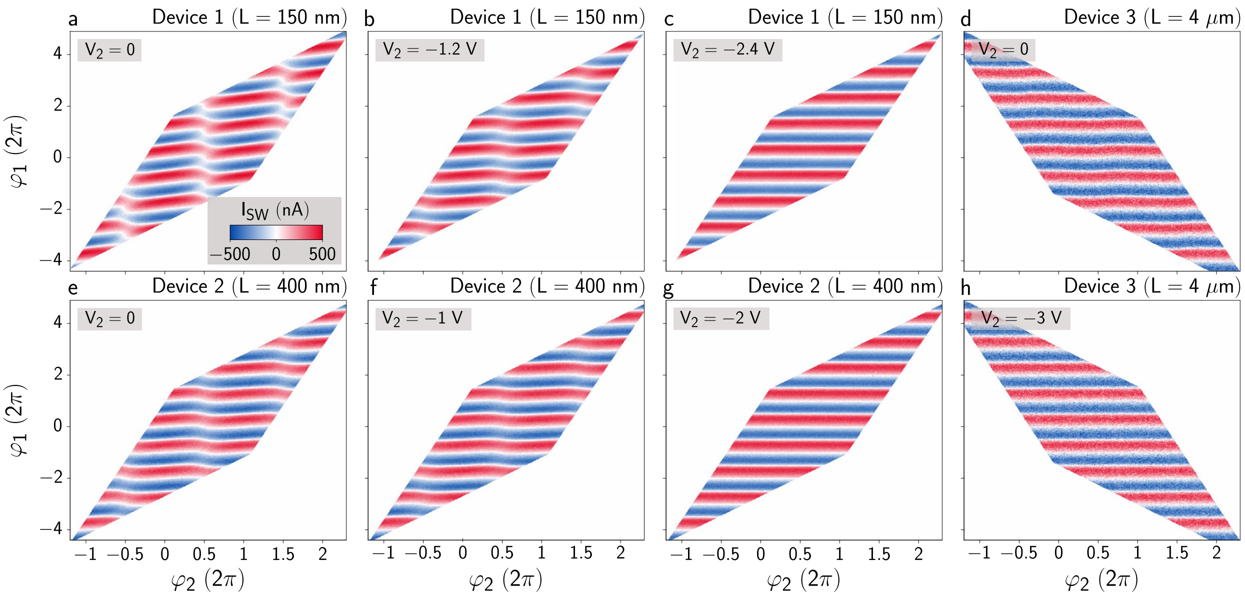

Figure S.1: Current-to-phase remapping of switching current measurements. (a-c) Switching current I_{SW} for Device 1, after subtracting the switching current of the Al constriction I_{Al}, for $V_1 = 0$ and $V_2 = 0$, -1.2 and -2.4 V, respectively. Data as in Fig. 2(a-c) of the Main Text, plotted as a function of the phase differences across JJ1 and JJ2, respectively φ_1 and φ_2. Flux line currents (I_L, I_R) were converted to phases (φ_1, φ_2) using Eq. 1, obtained from the periodicity directions (Φ_L, $\Phi_R - \Phi_L$). (d) Switching current I_{SW} of Device 3 for $V_1 = V_2 = 0$, in the (φ_1, φ_2) basis. (e-g) Same as panels (a-c) for Device 2, with $V_2 = 0$, -1 and -2 V respectively. (h) Switching current I_{SW} in Device 3 for $V_2 = -3$ V, in the (φ_1, φ_2) basis. All measurements shown in this figure were taken at $V_1 = 0$.

Linecuts of the data in Fig. S.1 along φ_1 are plotted in Fig. 4(a, d) of the Main Text, at $\varphi_2 = 0.8\pi$. The anomalous switching current ΔI_1, plotted in Fig. 4(b, e) of the Main Text, was obtained as a linecut of the data in Fig. S.1 along φ_2, for $\varphi_1 = 0$. To account for small misalignment of the $\Phi_L = \Phi_R = 0$ origin with respect to the data, a constant switching current offset was subtracted from each dataset such that the oscillations in ΔI_1 were symmetric. Anomalous phase shifts $\Delta\varphi_1$ were calculated from the $I_{SW} = 0$ position

where $\partial I_{SW}/\partial\varphi_1 > 0$, as a function of φ_2. Phase shifts $\Delta\varphi_1$ were calculated relative to the data where no current flowed through JJ2 ($V_2 = -2.4$ V for Device 1, $V_2 = -2$ V for Device 2). We expect a symmetric deviation in phase across a full period, so a small constant offset was independently obtained and subtracted from each dataset such that the oscillations in $\Delta\varphi_1$ were symmetric.

Measurements on Device 4

Measurements were performed on a fourth device, identical in design to Device 1 ($L =$ 150 nm). Switching current measurements of Device 4 are summarized in Fig. S.2, after subtracting a background corresponding to the switching current of the Al constriction, I_{Al}. The switching current I_{SW} was measured as a function of the current injected into the left and right flux lines, I_{L} and I_{R}. The currents $(I_{\mathrm{L}}, I_{\mathrm{R}})$ correspond to fluxes $(\Phi_{\mathrm{L}}, \Phi_{\mathrm{R}})$ threading the left and right loops, respectively. The phase difference across JJ1, φ_1, is expected to be modulated most strongly for fluxes threading both loops, i.e., $\Phi_{\mathrm{L}} + \Phi_{\mathrm{R}}$. In the case of no coupling between the JJs, the switching current is expected to be constant as a function of $\Phi_{\mathrm{R}} - \Phi_{\mathrm{L}}$. The phase difference across JJ2 is constant as a function of Φ_{L}. These directions are indicated on Fig. S.2(a) as the black arrows. When $V_1 = V_2 = 0$ [Fig. S.2(a)], there is a clear distortion of the switching current away from the phase axes, indicating hybridization with JJ2. From Fig. S.2(a), the size and shape of this distortion is qualitatively similar to that of Device 1 in the same gate configuration [Fig. 2(a) of the Main Text]. Figure S.2(b) shows the switching current as a function of phase differences across the JJs, (φ_1, φ_2), obtained using the same method outlined in Eq. 1. The transformation matrix $\mathbf{M}$ for Device 4 was:

$$\mathbf{M} = \begin{pmatrix} 0.64 & -1.98 \\ 6.23 & -3.00 \end{pmatrix} \text{ pH}, \tag{4}$$

very similar to Eqs. 2 and 3 for Devices 1 and 2.

Measurements were performed for different gate voltages V_2 applied to JJ2: $V_2 = -1$ V for Fig. S.2(c) and $V_2 = -3$ V for Fig. S.2(e). Figures S.2(d) and (f) show the switching current after transformation by matrix $\mathbf{M}$, for Figs. S.2(c) and (e) respectively. When JJ2 was partially depleted, but still allowed a current to flow, there was a distortion of I_{SW} but it was less pronounced than for $V_2 = 0$. For $V_2 = -3$ V, where no current could flow through the fully closed JJ2, there was no distortion of the switching current from the $\Phi_{\mathrm{R}} - \Phi_{\mathrm{L}}$ direction and oscillations in I_{SW} occurred with a single periodicity axis. In this configuration, there was no coupling between JJs and the current-phase relation (CPR) was that of JJ1 alone.

The anomalous switching current ΔI_1 at $\varphi_1 = 0$ is plotted in Fig. S.2(g) as a function of φ_2, for different gate voltages V_2 (see colors). A large, φ_2-dependent anomalous switching current was observed for $V_2 = 0$, which was smaller for $V_2 = -1$ V and absent for $V_2 = -3$ V. The phase shift $\Delta\varphi_1$ is quantified in Fig. S.2(h), as a function of φ_2 for different gate voltages V_2. The maximum phase shift for $V_2 = 0$ was $\Delta\varphi_1 = \pm 0.24\pi$ at $\varphi_2 = 0.8\pi$, almost identical to the result of Device 1. The size of the phase shift was smaller for more negative V_2, and completely suppressed when JJ2 was closed.

Figure S.3 presents the dependence of the switching current of Device 4 on the gate voltages V_1 and V_2. Figure S.3(a) shows I_{SW} as a function of flux line currents I_{L} and I_{R}, for $V_1 = -1.5$ V and $V_2 = 0$. While the oscillation amplitude was reduced relative to the $V_1 = 0$ configuration, the switching current modulation was comparable to that of Fig. S.2(a). The switching current was measured along the path γ, parallel to the $\Phi_{\mathrm{L}} + \Phi_{\mathrm{R}}$ direction. Figure S.3(b) shows I_{SW} along γ as a function of V_2, with $V_1 = 0$. Selected linecuts are shown in Fig. S.3(c). The position of $I_{\mathrm{SW}} = 0$ shifted as a function of V_2 and the oscillations changed from being distorted for $V_2 > -1.5$ V, to a regularly skewed CPR for $V_2 < -1.5$ V. This demonstrates the strong effect of V_2 on the CPR of JJ1. Figures S.3(d) and (e) show I_{SW} along γ as a function of V_1, for $V_2 = -3$ V and 0 respectively. The switching current had a conventional forward-skewed CPR in Fig. S.3(d), since no current flowed through JJ2. Decreasing V_1 only decreased the amplitude of oscillations. For $V_2 = 0$, a current flowed

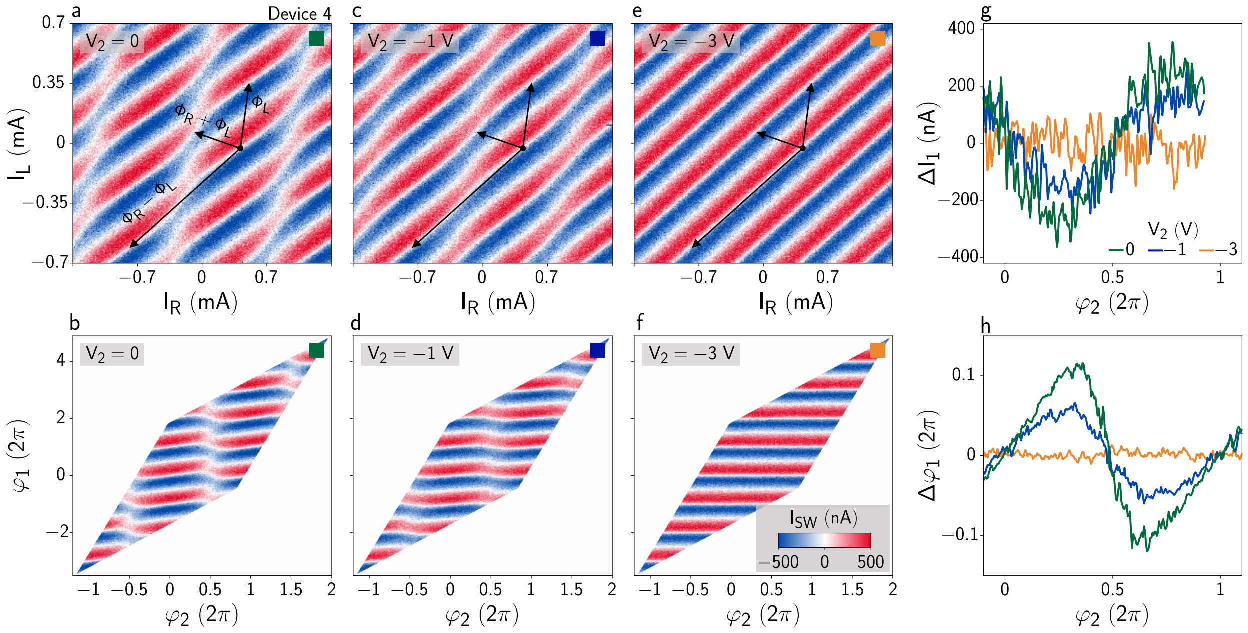

Figure S.2: Phase-dependent supercurrent in Device 4. (a, b) Switching current $I_{\rm SW}$ of Device 4, identical to Device 1 with a length of $L = 150$ nm, as a function of flux-line currents $(I_{\rm L}, I_{\rm R})$ and junction phases (φ_1, φ_2), respectively. Data is plotted after subtraction of the switching current of the Al constriction $I_{\rm Al}$, for $V_1 = V_2 = 0$. Black arrows indicate the direction $\Phi_{\rm L} + \Phi_{\rm R}$, which is the direction of maximum modulation of φ_1, and $\Phi_{\rm R} - \Phi_{\rm L}$, where φ_1 is constant and φ_2 is modulated. (c, d) Same as panels (a, b) for $V_2 = -1$ V. (e, f) Same as panels (a, b) for $V_2 = -3$ V. (g) Anomalous supercurrent ΔI_1 at $\varphi_1 = 0$, as a function of φ_2 for three values of V_2 [colors, corresponding to panels (b, d, f)]. (h) Anomalous phase shift $\Delta\varphi_1$ as a function of φ_2 for three values of V_2 [see legend in panel (g)].

through JJ2. Nevertheless, V_1 only caused a decrease in the overall amplitude of oscillations, without introducing distortions or phase shifts. This is also evident from selected linecuts, displayed in Fig. S.3(f), showing that V_1 had little to no effect on the anomalous phase shift $\Delta\varphi_1$.

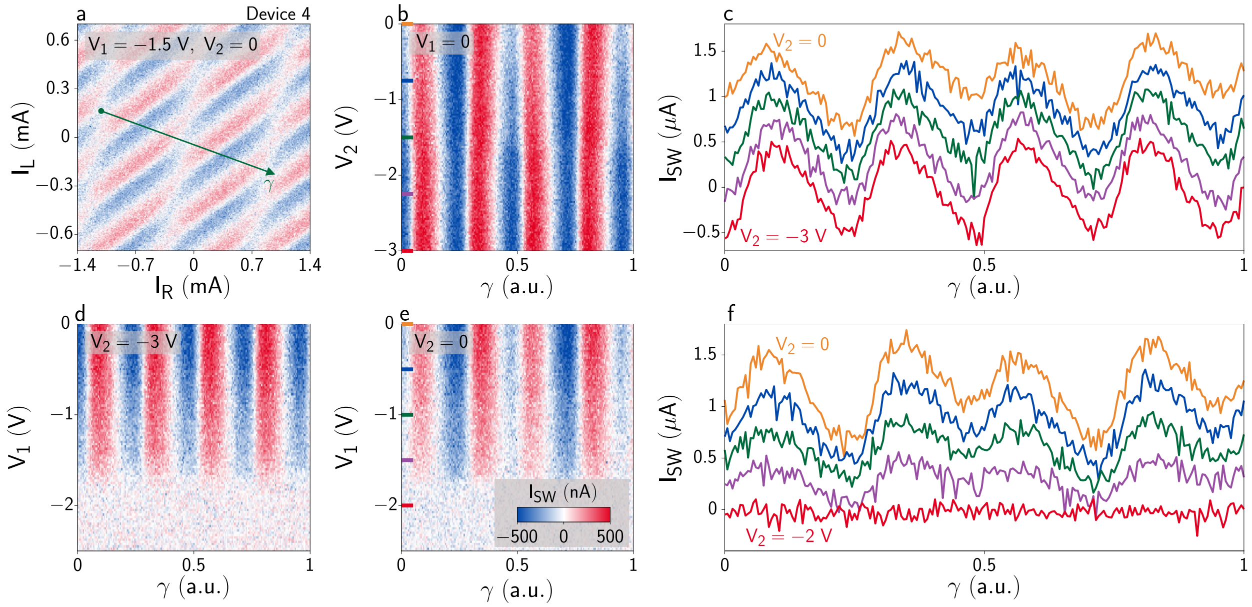

Figure S.3: Gate dependence of switching currents in Device 4. (a) Switching current I_{SW} for Device 4 as a function of flux line currents I_{L} and I_{R}, at $V_1 = -1.5$ V and $V_2 = 0$. Data is qualitatively similar to that of Device 1, in Fig. 3(a) of the Main Text. The path γ, parallel to the $\Phi_{\mathrm{L}} + \Phi_{\mathrm{R}}$ direction, is indicated by the arrow. (b) Switching current I_{SW} along γ, as a function of V_2 with $V_1 = 0$. In this configuration, the positions where $I_{\mathrm{SW}} = 0$ shift as V_2 is decreased. (c) Linecuts of I_{SW} extracted from panel (b), at different values of V_2 [indicated by the colored markers in panel (b)]. (d) Switching current I_{SW} along γ as a function of V_1, with $V_2 = -3$ V such that no current flows through JJ2. (e) As in panel (d), but measured with $V_2 = 0$. Also in this configuration, despite a current can flow through both JJs, there is no shift in the $I_{\mathrm{SW}} = 0$ position. (f) Linecuts of I_{SW} extracted from panel (e), at different values of V_1 [indicated by the colored markers in panel (e)].

References

(1) Coraiola, M.; Haxell, D. Z.; Sabonis, D.; Weisbrich, H.; Svetogorov, A. E.; Hinderling, M.; ten Kate, S. C.; Cheah, E.; Krizek, F.; Schott, R.; Wegscheider, W.; Cuevas, J. C.; Belzig, W.; Nichele, F. Hybridisation of Andreev bound

states in three-terminal Josephson junctions. *arXiv Preprint* **2023**, arXiv:2302.14535 https://arxiv.org/abs/2302.14535 (accessed 01.06.2023).

(2) Annunziata, A. J.; Santavicca, D. F.; Frunzio, L.; Catelani, G.; Rooks, M. J.; Frydman, A.; Prober, D. E. Tunable superconducting nanoinductors. *Nanotechnology* **2010**, *21*, 445202.

(3) Nichele, F.; Portolés, E.; Fornieri, A.; Whiticar, A. M.; Drachmann, A. C. C.; Gronin, S.; Wang, T.; Gardner, G. C.; Thomas, C.; Hatke, A. T.; Manfra, M. J.; Marcus, C. M. Relating Andreev bound states and supercurrents in hybrid Josephson junctions. *Phys. Rev. Lett.* **2020**, *124*, 226801.
